# Clinical evaluation of acute necrotizing encephalopathy in children

**DOI:** 10.3389/fped.2022.947693

**Published:** 2022-08-25

**Authors:** Xiaowei Fan, Li Huang, Suyun Li, Sida Yang, Yongling Song, Qinglian Chen, Yumei Xiong, Qiuyan Peng, Wencheng Ma, Dandan Hu, Peiqing Li

**Affiliations:** Guangzhou Women and Children’s Medical Center, Guangzhou Medical University, Guangdong Provincial Clinical Research Center for Child Health, Guangzhou, China

**Keywords:** acute necrotizing encephalopathy, children, brainstem auditory evoked potential, magnetic resonance imaging scores, acute necrotizing encephalopathy severity score, modified Rankin scale

## Abstract

**Objective:**

Acute necrotizing encephalopathy (ANE) is a rare but severe encephalopathy and is associated with a high morbidity and mortality. We aimed to analyze and compare the clinical features and predictive indicators of pediatric ANE.

**Materials and methods:**

This retrospective study included children with ANE diagnosed at Guangzhou Women and Children’s Medical Center between November 2018 and January 2020. Pediatric patients’ information, including clinical characteristics, laboratory tests, neuroelectrophysiology and brain magnetic resonance imaging (MRI) findings, MRI score, brainstem auditory evoked potential (BAEP) grades, ANE severity scores (ANE-SS), and modified Rankin scale (mRS), were collected.

**Results:**

Twelve ANE patients were included. Among them, one patient (8.3%) died from brainstem dysfunction, one (8.3%) recovered and 10 (83.3%) experienced neurological sequelae. All patients had an initial viral infection and neurological symptoms such as acute disturbance of consciousness (ADOC) or seizure, and the interval from onset of the disease to neurological manifestations was 3 (1.25–3) days. MRI score-I ranged from 1 to 3 (1.8 ± 0.7), MRI score-II ranged from 1 to 4 (2.5 ± 1.1). ANE-SS varied from 1 to 6 (3.9 ± 1.3). The scores of mRS were from 0 to 6 (2.9 ± 1.7). Higher MRI score were associated with worse outcomes, while the BAEP grade and ANE-SS score were not significantly associated with mRS.

**Conclusion:**

ANE is a severe encephalopathy syndrome with rapid progression, resulting in serious neurological sequelae. Compared with BAEP grade and ANE-SS, brain MRI shows more comprehensive advantages in predicting the prognosis of ANE patients. More in-depth research and better indicators are still needed to support the evaluation and treatment of ANE.

## Highlights

- Research background

Acute necrotizing encephalopathy (ANE) is one of the most severe neurological complications associated with acute viral infection, and its adverse sequelae rate is very high. Early detection and prognostic assessment of ANE are necessary to prolong survival.

- Research motivation

Children are more susceptible to respiratory viral infections and have a high risk of developing central nervous system complications. ANE is one of the most severe complications, and is associated with a poor prognosis. Most of the survivors have neurological sequelae. The early diagnosis and differential diagnosis of ANE is a current challenge. Thus, improving clinicians’ awareness and management of ANE can save lives of patients and improve their outcomes.

- Research objectives

To analyze the clinical characteristics of ANE patients and to find out the clinical predictive indicators with comprehensive advantages for ANE.

- Research methodology

From November 2018 to January 2020, the data of ANE children < 14-year-old were collected in this retrospective study. The clinical manifestations, laboratory test results, neuroelectrophysiology and imaging findings, treatment, and outcomes of ANE children were analyzed.

- Conclusion

ANE is an acute catastrophic encephalopathy that causes severe consequences. In children with convulsions accompanied by consciousness disorder after acute viral infection, the risk of ANE should be considered. More in-depth research and better predictive indicators are still demanded to support the evaluation and treatment of ANE.

## Introduction

Acute necrotizing encephalopathy (ANE) is a rare type of brain disease characterized by rapid onset of seizure and severe neurological complications such as altered consciousness changes following viral infections, which can lead to high mortality and disability (1). Proposed by Mizuguchi et al. (2) and Neilson et al. (3), ANE is diagnosed based on brain radiological imaging characteristics and laboratory findings. Typical radiographs show multiple symmetrical lesions in regions such as the thalamus, basal ganglia, and brain stem, which are key indicators of ANE. Laboratory examinations often demonstrate elevated aspartate aminotransferase (AST), alanine aminotransferase (ALT), and cerebrospinal fluid (CSF) protein levels, while ammonia level and CSF cell count were normal (4).

The prognosis of ANE patients is poor, with only 13% of patients recovering, 56% having permanent sequelae, and 28% dying (1). Yamamoto et al. (5) reported that shock, brainstem lesions, and > 48 months of age were high-risk factors for permanent neurological sequelae and death. Okumura et al. (6) found that ANE children who received steroid treatment within 24 h after the onset of symptoms achieved better prognosis, without signs of brainstem lesions.

Despite ANE being a rare condition, its dismal outcomes and urgent management have prompted researchers to identify the patients at high risk of poor prognosis as early as possible. Brainstem auditory evoked potential (BAEP) grades (7), magnetic resonance imaging (MRI) score (8), ANE severity score (ANE-SS) (5), and modified Rankin Scale (mRS) (9) have been used to stratify ANE risk. Nonetheless, the advantages and disadvantages of these scoring tools in assessing ANE have not been compared.

Therefore, in the present study, the clinical data of ANE children admitted at a tertiary pediatric hospital in China from November 2018 to January 2020 were collected and analyzed, and the clinical indicators of pediatric ANE was comprehensively evaluated.

## Materials and methods

### Study design and patients

This retrospective study included children with ANE who were diagnosed between November 2018 and January 2020 at the Emergency Department of Guangzhou Women and Children’s Medical Center, China. Ethical approval was obtained from the Ethics Committee of Guangzhou Women and Children Medical Center [Sui Fuer Kelun (2019) No. 38201]. All patients signed informed consent upon admission.

The diagnosis of ANE was based on the criteria established by Mizuguchi et al. (2). All included patients were evaluated by a pediatric neurologist and a pediatric radiologist, and presented with acute encephalopathy and typical bilateral thalamic lesions on imaging tests. The exclusion criteria were: (1) severe central nervous system conditions such as mass, tumor, traumatic cerebrovascular malformation, or pachygyria; (2) hereditary metabolic diseases; and (3) meningitis.

### Data collection and outcomes

The clinical data of children with ANE, including sex, age, clinical manifestations, blood and CSF tests, neuroelectrophysiology and brain imaging findings, treatments and outcomes, were collected from the hospital electronic medical record system (EMR). BAEP grades were determined according to the method described by Hall et al. (7). MRI scores (0–4 point scale) were calculated using the method developed by Wong et al. (8). A MRI score of one point represented hemorrhage, cavitation, brain stem and white matter involvement. MRI score-I was calculated based on the initial scan, and MRI score-II was measured based on the initial and follow-up scans. The prognosis of ANE was measured by ANE-SS (0–9 point scale) based on clinical findings (5), with 3 points for shock at onset, 2 points for brainstem lesions, 2 points for age older than 48 months, 1 point for platelet count less than 100,000/μl, and 1 point for cerebrospinal fluid protein greater than 60 mg/ml. All laboratory tests were performed within 24 h after admission. GCS was evaluated within 1–3 days after acute disturbance of consciousness (ADOC). ANE-SS was evaluated within 7 days after admission. MRI was performed using a super-conducting 3.0-T system with standard head coils (Skyra; Siemens Medical Solution, Erlangen, Germany). All pulse sequences were adopted as clinically indicated. The 6-month post-injury outcomes were evaluated using the mRS.

### Statistical analysis

Statistical analysis was performed using SPSS 26.0 (IBM, Armonk, NY, United States). Categorical variables were described as *n* (%). Continuous variables with a normal distribution were presented as means ± standard deviation (SD). Continuous variables without a normal distribution were presented as medians (interquartile range). Ranked variables were compared using Spearman’s rank correlation analysis. A two-sided *p*-value < 0.01 was considered statistically significant.

## Results

### Patient characteristics

Twelve children diagnosed with ANE were included in this study. Patients’ data including clinical characteristics, viral study, duration of mechanical ventilation, hospital stay, and outcomes were collected. The age of the children ranged from 11 months to 8 years, with a median of 43 months (IQR: 24.5, 51.75), and 83.3% (10/12) were younger than 5 years old. All patients had an initial viral infection presentation and neurological symptoms. Nine (75%) patients had respiratory symptoms (nasal discharge and cough), and five (41.7%) had gastrointestinal symptoms (vomiting and diarrhea). All pediatric patients presented ADOC or rapid seizure within 3 days after the onset of ANE. Six (66.7%) patients had a GCS < 8, while three (25%) were unable to be assessed due to sedation during mechanical ventilation. Eleven (91.7%) children had seizures, among whom 9 (75%) experienced more than three seizures or convulsions. Central respiratory failure occurred in seven (58.3%) children. Shock was noted in one (8.3%) child. Unequal pupil size was detected in two (16.7%) pediatric patients. All children were treated with high-dose gamma immunoglobulin combined with high-dose methylprednisolone within 1–3 days after admission. Nine (75%) children received mechanical ventilation for an average of 12.9 ± 6.2 days. The length of hospitalization stay was 28.5 ± 10.1 days. Except for one (8.3%) death from brainstem dysfunction and one (8.3%) recovery, the remaining 10 (83.3%) pediatric patients had neurological sequelae, including cognitive impairment, dystonia, and epilepsy. The clinical characteristics of the children with ANE are shown in Table 1.

**TABLE 1:** Patient characteristics.

Case	Sex	Age (m)	Symptoms of onset	Seizures	GCS score	Central respiratory failure	Shock	Anisocoria	Treatment			Hospital stays (day)	Outcome
									IVIG (g/kg)	MP (mg/kg)	Mechanical ventilation (day)		
1	F	51	Fever, cough, vomiting	1	5	–	–	–	2	10	11	26	Epilepsy
2	F	52	Fever, cough	> 3	5	+	–	+	2	10	15	18	Death
3	F	24	Fever, cough	> 3	/	+	–	–	2	10	4	17	Cognitive dysfunction
4	M	63	Fever, cough, hoarseness	Status epilepticus	13	–	+	–	2	10	6	33	Cognitive Dysfunction
5	M	12	Fever, cough	> 3	/	+	–	+	2	10	15	39	Muscular hypertonia
6	F	49	Fever, cough, vomiting	> 3	6	–	–	–	2	10	0	24	Muscular hypertonia
7	F	26	Fever, chills, vomiting	> 3	/	+	–	–	2	20	27	45	Muscular hypertonia
8	F	46	Fever, cough	> 3	4	+	–	–	2	10	10	30	Muscular hypertonia
9	M	11	Fever, erythema	1	6	+	–	–	2	15	13	26	Cognitive dysfunction
10	F	40	Fever, cough, vomiting	> 3	4	+	–	–	2	10	15	47	Muscular hypertonia
11	F	30	Fever	> 3	10	–	–	–	2	10	0	21	Epilepsy
12	F	96	Fever, vomiting, diarrhea	0	13	–	–	–	2	15	0	16	Complete recovery

GCS, Glasgow Coma Scale; /, not available; IVIG, intravenous immunoglobulin; MP, methylprednisolone.

Nasopharyngeal swab real-time reverse transcription-polymerase chain reaction (rRT-PCR) testing showed six (50%) cases with influenza A infection, three (25%) infected by influenza B, one (8.3%) infected by parainfluenza virus, and one (8.3%) infected by human herpesvirus 6, while one (8.3%) case showed no viral infection. Laboratory examinations showed high serum levels (above the upper limit of normal reference ranges) of aspartic acid transferase (AST), creatine kinase (CK), lactate dehydrogenase (LDH) and α-hydroxybutyrate dehydrogenase (HBDH) in all cases, high alanine aminotransferase (ALT) level in 7 (58.3%) cases, high creatine kinase (CK) level in 8 (66.7%) cases, high creatine kinase isoenzyme (CK-MB) level in 11 (91.7%) cases, and high CSF protein levels in 10 (83.3%) cases. Two cases were analyzed for RANBP2 mutations, and one of them showed positive result (Table 2).

**TABLE 2:** Laboratory examination results.

Case	ALT (7–40 U/L) *	AST (5–60 U/L) *	CK (45–39 U/L) *	CK-MB (0–29 U/L) *	LDH (159–322 U/L) *	HBDH (207–244 U/L) *	PCT (< 0.25 ng/ml) *	CSF pressure (< 280 mm H_2_O)	CSF leukocyte (0–5 × 10^6^/L) *	CSF protein (0.15–0.45 g/L) *	Nasopharyngeal rRT-PCR	RANBP2 testing
1	198	251	470	115	508	394	90	300	4	2.72	FA	/
2	23	88	4209	87	454	252	5.65	105	2	0.16	FA	/
3	35	108	206	42	548	332	0.565	105	3	0.64	FA	/
4	15	72	684	92	1165	1178	0.05	300	0	0.21	FB	/
5	456	730	16549	207	1933	1420	18.4	100	27	1.07	FA	/
6	89	94	305	58	332	269	7.68	140	1	0.88	FA	/
7	317	121	1553	45	521	444	0.534	300	2	3.9	FA	/
8	4950	2107	163	23	578	420	12.5	180	4	4.98	FB	–
9	22	443	703	36	364	318	0.1	130	31	2.33	HHV-6	+
10	13	100	101	61	705	623	0.352	310	3	0.55	FB	/
11	6801	13165	606	49	8576	2914	34.7	138	2	1.95	PIV	/
12	326	565	562	39	988	618	90	330	2	0.63	–	/
Mean	1103.8	1487.0	2175.9	71.7	1389.3	765.2	21.7	203.2	6.8	1.7		
SD	2268.4	3722.1	4663.4	50.4	2307.4	769.6	33.5	95.2	10.5	1.5		

ALT, alanine aminotransferase; *, the range of reference value; AST, aspartic acid transferase; CK, creatine kinase; CK-MB creatine kinase isoenzyme; LDH, lactate dehydrogenase; HBDH, α-hydroxybutyrate dehydrogenase; PCT, procalcitonin; CSF, cerebral spinal fluid; FA, influenza A; FB, influenza B; HHV-6, Human Herpes Viruses-6; PIV, parainfluenza virus.

### Neuroelectrophysiology, magnetic resonance imaging findings, and scoring of acute necrotizing encephalopathy cases

**FIGURE 1:**
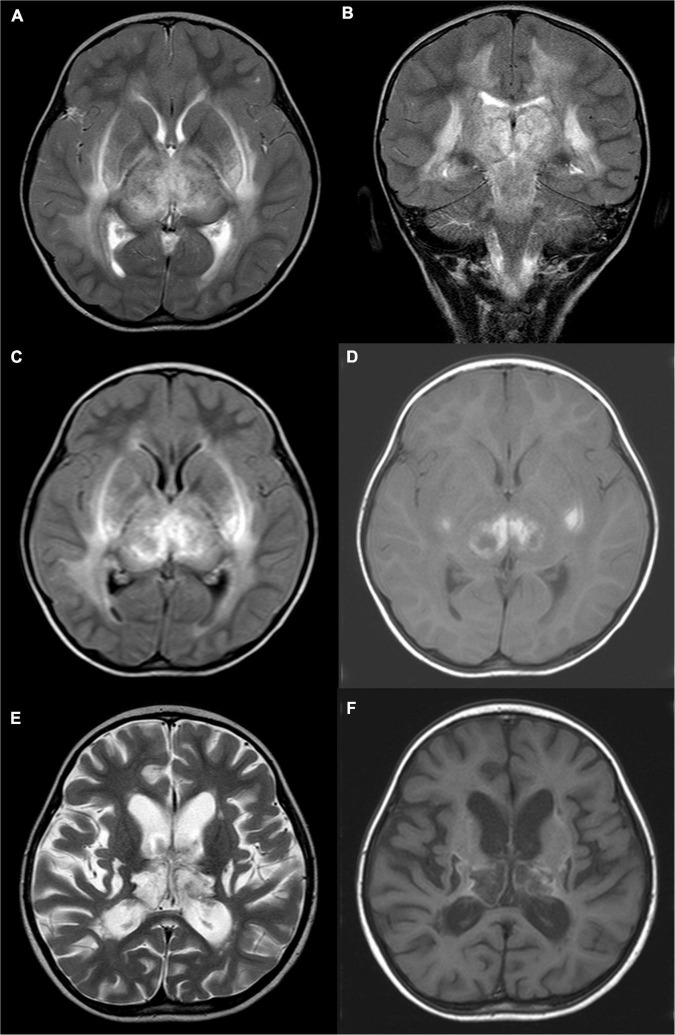
A 4-year-old girl with acute necrotizing encephalopathy, with fever for 1 day and coma. **(A)** T2WI cross-section shows slightly high signal intensity in bilateral thalamus, putamen, posterior limb of the internal capsule, external capsule, outermost capsule, and peripheral matter of posterior horn of lateral ventricle. **(B)** On T2WI coronal view, uneven, slightly high signal intensity is seen in the cerebral peduncle, midbrain, and dorsal pons. **(C)** T2WI water suppression cross-section shows slightly high signal intensity in bilateral thalamus, putamen, posterior limb of the internal capsule, external capsule, outermost capsule, and peripheral matter of posterior horn of the lateral ventricle. **(D)** T1WI plain cross-sectional shows patch-like hyperintensity in the bilateral thalamus and putamen. **(E)** Eight months later, T2WI cross-section shows irregular cystic hyperintensity in bilateral thalamus and putamen. **(F)** Eight months later, T1WI plain cross-sectional shows irregular cystic hypo-signal in bilateral thalamus and putamen.

**FIGURE 2:**
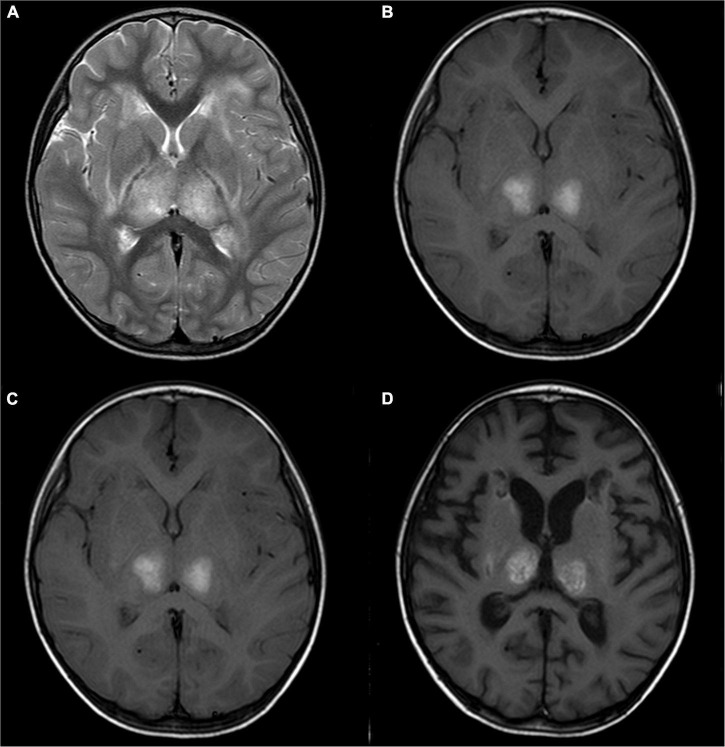
A 2-year-old girl with acute necrotizing encephalopathy, with fever and seizure. **(A)** T2WI shows slightly high symmetrical signals in the bilateral thalamus. **(B)** T1WI shows symmetrical hyperintensity in the bilateral thalamus. **(C)** One month later, T2WI shows irregular high signal in bilateral thalamus. **(D)** One month later, T1WI shows irregular high signal in bilateral thalamus.

The BAEP, EEG, and brain MRI anomaly rates were 81.8, 100, and 100%, respectively. Eleven pediatric patients underwent BAEP examination, and normal values were found in two children, and mild abnormalities were noted in nine children (showing prolongation of wave I–V latency). Ten (83.3%) pediatric patients showed diffuse slow waves on EEG and two (16.7%) showed asynchronous spike waves. Brain MRI displayed symmetric necrosis of the thalamus and other deep brain structures, particularly in the brain stem, peripheral white matter, and cerebellar medulla. All ANE children had thalamus lesions accompanied by cerebral and/or cerebellar lesions. Seven children (58.3%), including the non-survivors, developed brain stem lesions (Figures 1, 2 and Table 3). Eleven children underwent MRI more than twice, with a median interval of 116 days (IQR: 33, 273). Lesions with cavitation were found in five cases during follow-up. MRI score-I ranged from 1 to 3 (1.8 ± 0.7) points based on the initial scan. MRI score-II varied from 1 to 4 (2.5 ± 1.1) points based on the initial and follow-up scans. ANE-SS ranged from 1 to 6 (3.9 ± 1.3) points. The scores of mRS were from 0 to 6 (2.9 ± 1.7) points.

**TABLE 3:** Neuroelectrophysiology, MR imaging findings, and scoring of the ANE cases.

Case	BAEP grade	Brain MRI						MRI score-I	MRI score-II	ANE-SS	mRS
		Brainstem involve ment	Cerebral involve ment	Cerebellar involve ment	Basal ganglia involv ement	Intracranial hemorrhage	Cavitation				
1	2	+	+	+	+	+	+	3	4	5	4
2	2	+	+	+	+	+	–	3	3	4	6
3	2	–	+	–	–	–	–	1	1	3	1
4	1	–	+	–	+	–	–	1	1	5	2
5	2	–	+	+	+	+	+	2	3	1	4
6	1	–	+	+	+	–	+	1	2	3	1
7	2	+	+	+	–	+	+	2	4	5	4
8	2	+	–	+	+	–	–	2	2	5	2
9	2	+	+	–	–	+	+	2	4	3	4
10	2	+	+	–	–	–	–	2	2	4	3
11	2	+	–	+	+	+	–	2	3	6	4
12	/	–	–	+	–	–	–	1	1	3	0
Mean	1.8							1.8	2.5	3.9	2.9
SD	0.4							0.7	1.1	1.3	1.7

BAEP, brainstem auditory evoked potential; RANBP2, RAN binding protein 2; MRI, magnetic resonance imaging; MRI score-I was calculated based on the initial scan; MRI score-II was calculated based on both the initial and follow-up scans; ANE-SS, ANE severity scores; mRS, modified Rankin scale; /, not available; +, positive; –, negative; SD, Standard Deviation.

### Correlation analysis

The results showed that higher MRI score-I (*r*_*s*_ = 0.870, *p* = 0.000) and MRI score-II (*r*_*s*_ = 0.832, *p* = 0.001) were associated with worse outcomes (higher mRS), while BAEP grade and ANE-SS were not significantly associated with mRS (Table 4).

**TABLE 4:** Correlation analysis.

	mRS	
	r_*s*_	*P*
BAEP grade	0.550	0.080
MRI score-I	0.870	0.000*
MRI score-II	0.832	0.001*
ANE-ss	0.272	0.392

BAEP, brainstem auditory evoked potential; MRI, magnetic resonance imaging; MRI score-I was calculated based on the initial scan; MRI score-II was calculated based on both the initial and follow-up scans; RANBP2, RAN binding protein 2; ANE-SS, ANE severity scores; mRS, modified Rankin scale.

*p < 0.01.

## Discussion

Acute necrotizing encephalopathy is a fulminant encephalopathy that occurs after acute viral infection. It was first described in Japan in 1995 and was presumed to be related to race, but cases were eventually reported in many other countries, such as the United States and Spain, indicating that it was a global event (10). The majority of ANE patients are children, while a small number of adult cases have been reported. Being under 5 years of age is considered a factor associated with increased mortality and neurological sequelae (11). In the present study, 10 (83.3%) pediatric patients were younger than 5 years old, one died, and the rest had neurological sequelae. In addition, all children presented ADOC or rapid seizure in the early stages of ANE, which are regarded as high-risk factors for ANE development (12). Moreover, school-age children were also included in our study. The Satisfactory outcomes were achieved in case #12, an 8-year-old girl with typical ANE brain MRI changes but no convulsion. Nevertheless, due to the small number of cases, it was impossible to determine the prognostic role of age for ANE. Therefore, the influence of age on the occurrence and development of ANE needs to be further investigated by expanding the sample size.

The etiology and pathogenesis of ANE may be related to inflammatory responses and genetic variations. The common influenza A and B viral infections may lead to ANE, but parainfluenza viruses, human herpesvirus 6, and Coxsackievirus could also be causative factors of ANE (13). In some cases, a viral pathogen remains undetected (14). In our study, 76.9% of the ANE children were infected with influenza viruses, which was consistent with the results in previous literature. Okumura et al. (6) conducted a retrospective study of 22 ANE children, comparing influenza-positive versus influenza-negative ANE, and did not find significant differences in clinical characteristics, laboratory tests, imaging findings and outcomes between the two groups. Therefore, it was speculated that the pathogenic mechanism of ANE might not be related to the type of infected viruses, but to the activation of the host immune system after viral infection.

Song et al. (15) suggested that high CSF protein levels and serum PCT might be used as early predictive indicators for ANE. Yamamoto et al. (5) conducted a retrospective cohort study and found that age, CSF protein levels, and brainstem lesions were significantly associated with the prognosis of ANE, while the levels of AST, ALT, LDH and CK, and treatment modality were not significantly associated with ANE prognosis. In another small-size study, Appavu et al. (16) showed that age, CSF proteins, specific MRI lesion location, and EEG epileptiform discharge were not obviously correlated with ANE prognosis. Although similar to other studies, the present study showed elevated serum levels of PCT, AST, ALT, LDH, CK-MB, and CSF protein in most pediatric patients, but we could not determine the relationship between these indicators with the prognosis of ANE. In our cohort, two children developed mild CSF leukocytosis. However, we didn’t eliminate the two cases from the cohort because they showed typical clinical manifestations and imaging findings of ANE. We reviewed a large number of literature and there was evidence showing that some ANE patients could experience CSF leukocytosis (17). We speculated that some ANE children may have viral encephalitis at the same time.

RANBP2 gene mutations have been detected in familial and recurrent ANE patients (18). Two cases in our study were subjected to genetic mutation test, and one case was confirmed to carry RANBP2 spontaneous gene mutation with positive HHV-6B (relative abundance 94.4%) in CSF samples (19). Previous reports have shown that patients with ANE carrying the RANBP2 gene mutations usually suffer from infections at the beginning of their illness, which are caused by influenza virus, parainfluenza and Bocavirus, *Mycoplasma pneumoniae*, and SARS-CoV-2, suggesting that the infection by different pathogens might be a trigger factor for ANE onset in patients with RANBP2 gene mutations (20, 21). RANBP2 gene mutations are generally thought to be limited to patients with Caucasian ethnicity. Recently, several ANE patients with RANBP2 gene mutations have been reported in Asian populations (22), suggesting that race may not be a significant associated factor for the occurrence of ANE. It has also been reported that carnitine palmitoyl transferase II polymorphism is linked with the pathogenesis of ANE (23). More causative genetic mutations may be discovered in the future. The contributions of viral infection and genetic mutation to the pathogenesis of ANE deserve further research.

Presently, there are no specific treatments for ANE, but glucocorticoid, immunoglobulin, and related support therapy are used widely. Okumura et al. (24) considered that early use (within 24 h of onset of ANE) of high-dose glucocorticoid (30 mg/kg/day methylprednisolone for 5 days) was associated with a good prognosis of ANE without brainstem lesions. All pediatric patients in the present study were treated with immunoglobulin and glucocorticoid, but there was one death and 10 cases had residual neurological sequelae. Therefore, whether other mechanisms affect the effectiveness of the treatment remains to be determined. Li et al. (25) considered that the high mortality and neurological sequelae after immunoglobulin and steroid administration might be related to the lack of early detection of cerebrovascular microembolism/infarction and subsequent treatments. Therefore, the pathogenesis and treatment of ANE still need more thorough study.

A previous study reported that about 30% of ANE patients died, 10% recovered completely, and 60% had different degrees of neurological sequelae (26), similar to the rate of neurological sequelae of 83.3% in the present study. Therefore, early prognostic assessment to identify survivors is necessary. In the present study, mRS was used as a tool to evaluate the outcomes of ANE children. ANE-SS was applied as an index to evaluate the severity of ANE but does not contribute to the early diagnosis of ANE. Moreover, MRI scores were measured twice. MRI score-I was calculated based on the first scan and MRI score-II was calculated based on both the initial and follow-up scans. We found that both higher MRI score-I and MRI score-II were associated with worse mRS, while ANE-SS and BAEP were not significantly associated with mRS. The evaluation of MRI score-I was almost within the same time frame as the ANE-SS measurement. Moreover, the early diagnosis of ANE depends on changes in imaging parameters, such as the detection of cerebral thrombosis, cerebral hemorrhage, etc., which is also the basis for adjusting treatment strategies (27). Therefore, MRI is an important tool for the diagnosis and differential diagnosis of ANE, which help to establish the management strategy for ANE patients. However, there are some limitations in brain MRI detection, such as the risk of examination for patients in critical conditions. In our study, the ANE children required sedation during the examination, which further added to the difficulty of examination. At the same time, we believed that ANE-SS, as a comprehensive predictive indicator, had its advantages in judging the severity of the ANE disease. BAEP might play an auxiliary role in predicting the rehabilitation prognosis of brain injury (28), especially for children with brainstem symptoms who have not been given early diagnosis of ANE.

There were some limitations in this study. It was a retrospective single-center study with a very limited number of patients, and selection bias and memory bias might exist. For hematological index measurement, only values within 24 h after admission were considered, and the complete change trend was not evaluated. Finally, since children with ANE were in the neurodevelopmental stage, it is necessary to check the follow-up outcomes for a longer period of time.

## Conclusion

Acute necrotizing encephalopathy is an acute catastrophic encephalopathy that causes severe consequences. In children with convulsions accompanied by consciousness disorder after acute viral infection, the risk of ANE should be considered. More in-depth research and better predictive indicators are still demanded to support the evaluation and treatment of ANE.

## Data availability statement

The original contributions presented in this study are included in the article/supplementary material, further inquiries can be directed to the corresponding author.

## Ethics statement

The studies involving human participants were reviewed and approved by the Ethics Committee of Guangzhou Women and Children Medical Center. Written informed consent to participate in this study was provided by the participants’ legal guardian/next of kin.

## Author contributions

PL and XF conceived and designed the research. XF, LH, and QC collected the data. YS, QP, and YX made a statistical analysis. LH, SL, SY, WM, and DH made a clinical analysis. XF wrote the articles. All authors contributed to the article and approved the submitted version.
